# Using impression data to improve models of online social influence

**DOI:** 10.1038/s41598-021-96021-3

**Published:** 2021-08-16

**Authors:** Rui Liu, Kevin T. Greene, Ruibo Liu, Mihovil Mandic, Benjamin A. Valentino, Soroush Vosoughi, V. S. Subrahmanian

**Affiliations:** 1 Department of Computer Science, Dartmouth College, Hannover, 03755 USA; 2 Department of Government, Dartmouth College, Hannover, 03755 USA

**Keywords:** Computer science, Information technology

## Abstract

Influence, the ability to change the beliefs and behaviors of others, is the main currency on social media. Extant studies of influence on social media, however, are limited by publicly available data that record expressions (active engagement of users with content, such as likes and comments), but neglect impressions (exposure to content, such as views) and lack “ground truth” measures of influence. To overcome these limitations, we implemented a social media simulation using an original, web-based micro-blogging platform. We propose three influence models, leveraging expressions and impressions to create a more complete picture of social influence. We demonstrate that impressions are much more important drivers of influence than expressions, and our models accurately identify the most influential accounts in our simulation. Impressions data also allow us to better understand important social media dynamics, including the emergence of small numbers of influential accounts and the formation of opinion echo chambers.

## Introduction

Since 2005, the number of American adults using social media has risen from 5 to 72%. Social media now surpasses print newspapers as a source of news for Americans. Similarly, spending on digital advertising in the United States now exceeds spending on all other forms of advertising combined, with Facebook alone accounting for nearly a quarter of those dollars. Much of the activity on social media is designed to influence users’ beliefs and behaviors. Reports of organized online social influence (OSI) operations are now widespread and include both “good” influence operations as well as malicious ones. “Good” influence operations include campaigns focused on wellness such as the Healthy Together Victoria campaign in Australia^1^ and the state of Maryland’s social media campaign to promote mask usage^2^. Malicious influence operations include allegations of foreign state-backed influence operations in the 2016 US election^3^, anti-vaccination campaigns^4^, and COVID-19 related misinformation^5^. Understanding how influence occurs on social media is, therefore, of utmost importance for almost every aspect of modern society. This has led to an explosion of research on online social influence from computer science^6–9^, physics^10,11^, and the social sciences^12,13^.

Yet, almost all these studies have important limitations: (1) due to their reliance on publicly available data from social media platforms, extant models of influence rely exclusively on expression data, that is, data about the active engagement of users with content. For instance, on Twitter, this corresponds to tweets, likes, retweets, and making follower/friend links. Notably, data about exposure to content, also known as impression data, is missing in these studies. For example, a user may passively view a post on their home page without registering a click. While this type of data can be viewed by Twitter account owners, it is typically not available across users. (2) Moreover, these studies typically have no way to determine the ground truth data about exactly whose opinions or behaviors (other than expressions) were influenced and to what extent. These limitations make it difficult to tune and evaluate models of social influence. (3) Finally, few existing studies have been able to systematically study the goals, strategies and effects of OSI operations because doing so requires positive control of the OSI operation by the researcher as part of the study design. This is difficult using observational data because it frequently violates the terms of use of social media platforms and/or raises ethical issues regarding performing experiments on human subjects without consent. While previous work has used the click history of users to study impressions on social media, they also face similar limitations. These studies have access only to the actions and impressions of select users, not of the broader network of users that they interact with. Our research design allows for complete access to information on every user’s expressions and impressions.

To overcome these limitations, we designed and ran a social media simulation (with IRB approval) using an original, web-based micro-blogging platform that captures much of Twitter and Facebook’s functionality. The simulation recruited a set of 287 users to use the platform over the course of 5 days. 200 users were instructed to run operations designed to influence the opinions of other users on 8 relatively current and controversial subjects (e.g., Is the US doing enough to combat COVID-19? Are foods containing genetically modified ingredients safe and healthy to eat?). A separate group of 87 observers, who were only passively observing the conversations on the platform, were surveyed before the simulation and at the end of each day for their opinions on those 8 topics. These users were also asked to identify the accounts whose posts most influenced them. Importantly, in addition to commonly used expression data, the software platform also captured impression data, which consists of the posts that users viewed, but did not actively engage by liking, re-posting, commenting, or following. Although users knew they were participating in a simulation, most agreed that the experience faithfully represented social media use in the real world. At the end of the simulation, 86% of subjects reported that the platform was somewhat or highly realistic. Further details regarding the simulation are described in the methods section.

We make two major contributions. First, we define the novel concepts of direct influence networks (DINs) and full influence networks (FINs) that trace back the impression or expression events that precede an influence event. We propose three influence models, FI1 , FI2 , FI3 that leverage both expression and impression data and build on FINs to create a more complete picture of the mechanics of social influence. The second set of contributions involve 5 novel findings. (1) We demonstrate that impressions account for far more exposure to information on social media than do expressions and that impressions and expressions are not highly correlated. Yet, for the reasons described above, impressions have been largely ignored by the majority of the literature to date. (2) We find that once participants adopt a position on a particular subject, the diversity of positions on the same subject they choose to view (as measured by entropy) decreases dramatically over time. This suggests the potential for influence operations to polarize social media users through the formation of echo chambers. Though the existence of echo chambers has been described before^14,15^, to the best of our knowledge, we are the first to document the emergence of echo chambers using both impression and expression data from a social platform. (3) Our proposed models of influence accurately capture acts of successful influence in the ground truth generated by our simulation. (4) As expected, only a small minority of accounts were identified by observers as being influential. However, we found that accounts that users did not consider influential at the beginning of our study remained largely un-influential throughout the study, while users who were influential at the end of the study were likely to have been identified as influential from early on. (5) Using quasi-Poisson and Gamma-Poisson regression models to identify the determinants of influence, we found that all expression types (posts, comments, likes) and impressions are individually statistically significant and positively linked to influence at the $\hbox {p} < 0.01$ level. When we consider all these features simultaneously, however, only the impressions are positively linked and significant. Specifically, our regressions show that when the impressions of a particular account on the platform increase by one standard deviation, the expected number of times users cite the account as influential more than doubles. These results suggest that the single biggest determinant of influence on social media platforms is impressions, a factor that has hardly been studied in the growing OSI literature.

## Results

### Modeling influence

We say that an observer *o* was influenced at time *t* if they nominated a given user as being among the most influential accounts for that period of time. We call these *influence events*. An influence event *ie* occurs when an observer *o* expresses that a given user was among the most influential posters on a given day. To measure how an observer was influenced to nominate a given user we developed two kinds of networks that “traceback” the activities of observers, *direct influence networks*, and *full influence networks*.

#### Direct influence network

**Figure 1:**
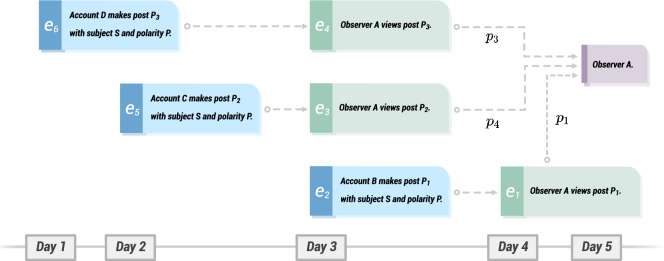
Sample direct influence network (DIN) for subject S with polarity P. This sample DIN is associated with an observer A (purple node) at the beginning of day 5. On day 4, there was an impression event (node $e_1$) in which that observer was served a post $P_1$ by account B. Thus, observer A may have been influenced by post $P_1$. Three events are reported on day 3 and raise interesting questions. Should account B (that posted post $P_1$ on day 3, see event $e_2$) receive credit for influencing A?.

Figure 1 shows a sample *Direct Influence Network* of observer A at the beginning of day 5 (purple node). In this example, we show the case when $\Delta =4$, tracing back to day 1. Though we use $\Delta =1$ in our experiments, the framework in this paper applies to any selected $\Delta .$

**Figure 2:**
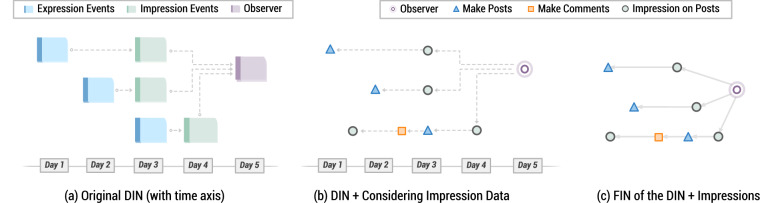
From DIN to FIN. (**a**) A duplicate of Fig. 1 showing the DIN. (**b**) Constructing FIN from DIN. (**c**) Complete sample FIN.

Suppose ${\mathsf {PIE}}(ie)$ denotes the set of possible impression and expression events for a certain subject *s* and polarity $p_t$ during the time interval $[t-\Delta , t]$ that could possibly have caused the influence event *ie* at time *t*. This is the set of all such events *e* such that there exists a time $t'\in [t-\Delta ,t]$ such that:

$$\begin{aligned}{ impression(e, s, p_t)=o\, \& \, (s,p_t)\in Expressions(e)} \end{aligned}$$

i.e. *e* is an impression event that was served to observer *o* by DartPost at time *t* connected to expression events under subject *s* and polarity $p_t$.

The *direct influence network* associated with an influence event *ie*, denoted ${{\textsf {DIN}}(ie)}$ is a network whose vertices include all events in ${\mathsf {PIE}}(ie)\,\cup \{ie\}$. The graph contains an edge from the events in ${\mathsf {PIE}}(ie)$ to *ie*.

#### Full influence network

As Fig. 1 shows, the direct influence network associated with the vertex $e_0$ only consists of the vertices $e_0,e_1,e_3,e_4$ because these are the vertices that capture posts/comments that were served up to A. However, we can “extend” node $e_4$ for instance, to encompass an associated direct influence network—in this case consisting of the vertices $e_4,e_6$ and the edge from $e_6$ to $e_4$. Thus, for an arbitrary event $e'$, we can define the Direct Influence Network ${{\textsf {DIN}}(e')}$ to include nodes corresponding to the elements of ${\mathsf {PIE}}(e)$ and expand the Direct Influence Network of an influence event *ie* by recursively expanding each vertex in ${{\textsf {DIN}}(ie)}$, then expanding out the new vertices iteratively until we either run out of the time window $[t-\Delta ,t]$ or reach a fixed point. The resulting network is called the *Full Influence Network* ${\mathsf {FIN}(ie)}$ associated with *ie*. Formally, we define:

$$\begin{aligned} {\textsf {FIN}}_0(ie)= & {} {\textsf {DIN}}(ie).\\ {\textsf {FIN}}_{j+1}(ie)= & {} \bigoplus _{x\in Vertices({\textsf {FIN}}_j(ie))}({\textsf {FIN}}_j(x)). \end{aligned}$$

where the $\oplus$ operator takes two graphs $(V,E),(V',E')$ as input. $\oplus (G,v)=(V\,\cup \, V',E\,\cup \, E',\wp \odot \wp ')$.

We start with the direct influence graph of *ie* (${\textsf {FIN}}_0$), which is the event of interest and expand it to include the direct influence graphs of all vertices in ${\textsf {DIN}}(ie)$ to get ${\textsf {FIN}}_1$. The same process is repeated till we eventually find a *k* such that ${\textsf {FIN}}_k(e')={\textsf {FIN}}_{k+1}(ie).$ Because the set of impressions and expressions in our data is always finite, such a *k* must exist. We denote this by $F_\infty (ie)$ for the sake of simplicity.

Figure 2 presents a simple example of the expansion of a DIN to a FIN. Subplot (a) duplicates Fig. 1 the DIN. In subplot (b) we see a new node (the orange square). This represents a comment made on a particular post. This content is included in the larger FIN as we expand outward and add nodes that are connected to nodes in the existing DIN. In this case, the comment is connected to a post in the DIN. In our simple example, the full expansion to the FIN adds only a few additional nodes and edges, but in the real network, this recursive expansion results in much larger and more complex networks than any single DIN. In subplot (c) we see the FIN after creating the edges outlined in subplot (b).

There are multiple ways in which we can define the full influence of an account *a* on an influence event *ie*. We now describe three such metrics below.

##### Network centric influence

Our first method to define the *full influence of an account* *a* *on the influence event* *ie* uses a weighted version of the full influence network. Given an edge (*u*, *v*) in this network, we define

$$\begin{aligned} {{{\mathsf {F}}}{{\mathsf {I}}}}_1(a,ie)= & {} \Sigma _{v\in {\textsf {FIN}}_\infty (ie)\, \& \, Account(v)=a}\, WPR(v) \end{aligned}$$

where *WPR* is the weighted PageRank function.

According to this metric, the full influence of an account *a* on *ie* is obtained by looking at all event nodes authored by *a* in the full influence graph associated with *ie* and adding up their weighted PageRanks^16^.

##### Temporal influence

The network centric model, however, cannot account for the expectation that the time at which an impression occurs is likely to affect how influential it is on a user’s opinion at some later time. For instance, the longer in the past an observer viewed a post, the more likely it is the post will be forgotten or superseded by other impressions. Users might also be more likely to be influenced by the first posts they viewed on a particular topic. For a given *ie*, we use the following notation:

$$\begin{aligned} MIN(ie)= & {} \mathsf {MIN}\{Time(v)\,|\, v\in {\textsf {FIN}}_\infty (ie)\}\\ MEAN(ie)= & {} \mathsf {AVG}\{Time(v)\,|\, v\in {\textsf {FIN}}_\infty (ie)\}\\ SD(ie)= & {} {{\mathsf {S}}}{{\mathsf {D}}}\{Time(v)\,|\, v\in {\textsf {FIN}}_\infty (ie)\} \end{aligned}$$

where the functions MIN, AVG and SD on a set denote the minimum, mean, and standard deviation of a set. We can define a new influence metric that takes “age” of an event into account when trying to assert its influence on *ie*.

$$\begin{aligned} {{{\mathsf {F}}}{{\mathsf {I}}}}_2(a,ie)= & {} \Sigma _{v\in {\textsf {FIN}}_\infty (ie)\, \& \, Account(v)=a}\, e^{\frac{|Time(v)-Mean(ie)|}{SD(ie)}}. \end{aligned}$$

Intuitively, ${{{\mathsf {F}}}{{\mathsf {I}}}}_2$ assigns greater importance to a user’s first impression on a subject as well as to his most recent impressions—but less importance to those that occurred somewhere in the middle.

##### Network and temporal influence

A third influence model considers a linear combination of the network centric and temporal influence metrics.

$$\begin{aligned} {{{\mathsf {F}}}{{\mathsf {I}}}}_3(a,ie)= & {} \Sigma _{v\in {\textsf {FIN}}_\infty (ie)\, \& \, Account(v)=a}\, e^{\frac{|Time(v)-Mean(ie)|}{SD(ie)}}*PageRank(ie). \end{aligned}$$

#### Capturing influence

Our ground truth influence data allows us to directly evaluate the accuracy of the FINs in capturing influence in our simulation. The ground truth influence data consists of the observers’ daily survey mentions of accounts they deemed influential. We are not aware of past efforts where ground truth is available about which accounts influenced which other accounts—for instance^6,7,17^, do not have ground truth assessments of who influenced a user to behave in a certain way.

At the end of each day, we record the ${{{\mathsf {F}}}{{\mathsf {I}}}}{}$ for account. We also record the list of accounts indicated as most influential by each observer. We evaluate the effectiveness of ${{{\mathsf {F}}}{{\mathsf {I}}}}$ at capturing ground truth influence by measuring the precision at K between the ranks of accounts (based on counts of mentions as being influential). The reported precision at K is computed as $\frac{\Sigma _{a,j}FI_j(a) < K}{\Sigma _{a,j} 1}$. For each K and each nomination *j* of participants named as being influential by observer *a*, we investigate the FINs of the observer *a* for all the subjects and polarities (positive and negative), and then compute the Full Influence of *j* as ${{{\mathsf {F}}}{{\mathsf {I}}}}_j(a) = MIN({{{\mathsf {F}}}{{\mathsf {I}}}}_j(a, s, p))$. Table 1 shows the results of our precision at K assessments for ${{{\mathsf {F}}}{{\mathsf {I}}}}_3$. We also performed a grid search with various parameter values for the damping factor $\delta$ used in Pagerank and show the results. We found that the results are not very sensitive to the choice of the damping factor and that the generally agreed-upon damping factor of 0.85^18^ produces acceptable results. ${{{\mathsf {F}}}{{\mathsf {I}}}}_1$ and ${{{\mathsf {F}}}{{\mathsf {I}}}}_3$ both achieve over 77% precision at $K=10$, i.e. when the top 10 accounts are compared. We only show the results for ${{{\mathsf {F}}}{{\mathsf {I}}}}_3$ in Table 1—similar results for ${{{\mathsf {F}}}{{\mathsf {I}}}}_1,{{{\mathsf {F}}}{{\mathsf {I}}}}_2$ are shown in the Supplementary Material 1.

In other words, our influence metrics accurately identify influential accounts in our simulation (according to the ground truth provided by observers) even when we focus only on the few most influential accounts. This is important, since we expect the distribution of influence on social media to be highly skewed^19–21^.

**Table 1:** Full precision at K results for ${{{\mathsf {F}}}{{\mathsf {I}}}}_3$.

Damping	K								
	1	5	10	20	30	40	50	100	116
0.85	0.228	0.620	0.783	0.794	0.794	0.794	0.794	0.794	1.000
0.90	0.228	0.620	0.783	0.794	0.794	0.794	0.794	0.794	1.000
0.70	0.250	0.620	0.783	0.783	0.794	0.794	0.794	0.794	1.000
0.50	0.239	0.630	0.783	0.783	0.794	0.794	0.794	0.794	1.000
0.30	0.239	0.641	0.772	0.783	0.794	0.794	0.794	0.794	1.000
0.10	0.217	0.630	0.772	0.783	0.794	0.794	0.794	0.794	1.000

### Impressions vs expressions

As described above, one of the key advantages of our study is our ability to collect impression data. Our simulation confirms that impressions are by far the most frequent type of user activity in social networks. Consequently, they also dominate the interactions on our social influence models. In fact, in our simulation, there were 35.6 times as many nodes in FINs that include both impressions and expressions. Figure S10 in the Supplemental Information 1 depicts an example based on data from our simulation of a full $\mathsf {FIN}$ and a $\mathsf {FIN}$ that is restricted to expression events only for the same influence event. The expression only model provides a much sparser picture of user interactions than the model including impressions.

To further illustrate the importance of impression data in influence modeling, we conduct a series of statistical analyses to investigate how both expression and impression data are associated with influence. Our measures of expressions consist of the number of posts, comments, and likes issued by a given user, while our impression data consists of the number of views a user receives. Our outcome variable records the number of times a given user is mentioned as being influential in the survey responses of the other participants in our simulation. Our independent and dependent variables are all aggregated to the user level.

Our statistical models are estimated using two generalizations of the Poisson distribution because our outcome variables are overdispersed count data. Overdispersion is confirmed using a $\chi ^2$ test. The first, the Quasi-Poisson, relaxes the assumption of the Poisson that the mean and variance are equal, instead assuming that the variance is a linear function of the mean^22,23^. This results in coefficient estimates identical to the Poisson, but allows for the standard errors to be adjusted based on the dispersion statistic estimated from the model. The second, the gamma-Poisson or Negative Binomial, allows the rate parameter of the Poisson distribution ($\lambda$) to be gamma distributed, providing additional flexibility to account for overdispersion^23,24^.

Table 2 shows the results of our two models. The results of the Quasi-Poisson model indicate that an account’s received (i.e. shown) views are positively and significantly associated with the number of times they are mentioned as being influential. The coefficients for the expression variables (Issued Posts, Issued Comments, Issued Likes) are either insignificant, or only marginally significant in the case of Issued Comments. In the gamma-Poisson model, we see similar results. Here, only the coefficient for Received Views is significant at conventional levels, while all of the expression measures are insignificant. To provide additional context for the effect size of Received Views, we first calculated the expected counts of expressed influence holding all of our independent variables at their mean values, finding a value of 1.91 for the Quasi-Poisson and 1.52 for the gamma-Poisson. We then increase the value of Received Views by one standard deviation and recalculate the expected counts, which increases to 4.3 in the Quasi-Poisson model and 5.1 in the gamma-Poisson model. In both models the expected number of mentions more than doubles. *In sum, we find that only impression based data is consistently positive and significantly associated with influence, based on our ground truth measure.* This further suggests that explanations of online influence based only on user expressions may be incomplete, and suggests that continued study on the role of impressions may provide novel insights into influence on social networks.

**Table 2:** Impact of user features on the number of times a user is named as an influential user by other users in the simulation.

	Dependent variable: expressed influence	
	Quasi-Poisson	Gamma-Poisson (NB)
Issued posts	− 0.012	− 0.009
	(0.011)	(0.015)
Issued comments	0.012$^{*}$	0.012
	(0.006)	(0.009)
Issued likes	0.008	0.0003
	(0.007)	(0.010)
Received views	0.0002$$^{***}$$	0.0003$$^{***}$$
	(0.00003)	(0.00004)
Constant	0.007	$$-\,0.554^{**}$$
	(0.257)	(0.275)
Observations	82	82

$$^{*}\hbox {p}<0.1$$; $$^{**}\hbox {p}<0.05$$; $$^{***}\hbox {p}<0.01$$.

### Influence operations and formation of echo chambers

We found that as the simulation continued, users increasingly engaged with less diverse opinions across subjects. At the beginning of the simulation, observers were exposed to different positions (supporting/opposing) for each subject, but over the course of the simulation, they increasingly viewed content that primarily expressed a single position.

**Figure 3:**
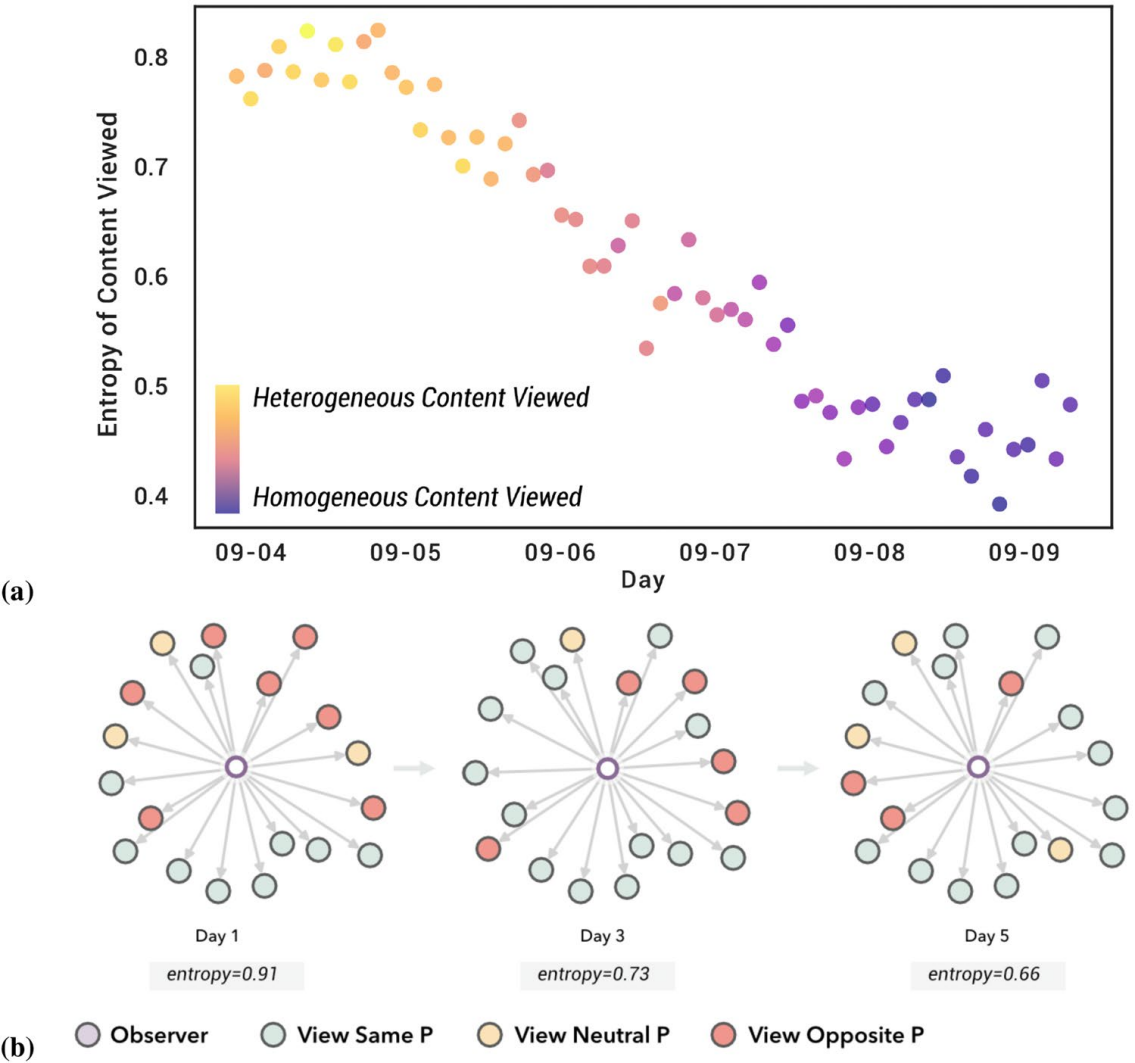
(**a**) Changes in entropy of posts/comments served to observers aggregated across observers. (**b**) Change in entropy for a sample direct influence network over time. This shows a specific example in which the entropy of each node in the immediate ${{\mathsf {DIN}}}$ around the user gets progressively more homogeneous as the simulation proceeds (mostly holding the same view as the observer in the example.

We quantified diversity using the entropy of the polarity of posts in DINs. Figure 3b shows an example of the DINs of an observer for the same subject over different days and the change in the entropy of the polarity. As the diversity of polarity decreased, the entropy increased.

Figure 3a shows the overall decrease in entropy for all observers over the 5 days of the simulation. The figure suggests the formation of echo chambers, where observers, quickly start filtering out content with the opposing polarity and mainly focus on content that reinforces their initial position.

These findings illustrate how quickly echo chambers can be formed through influence operations. The speed at which these echo chambers are formed is also surprising, given the short duration of this simulation.

### Emergence and persistence of influential accounts

**Figure 4:**
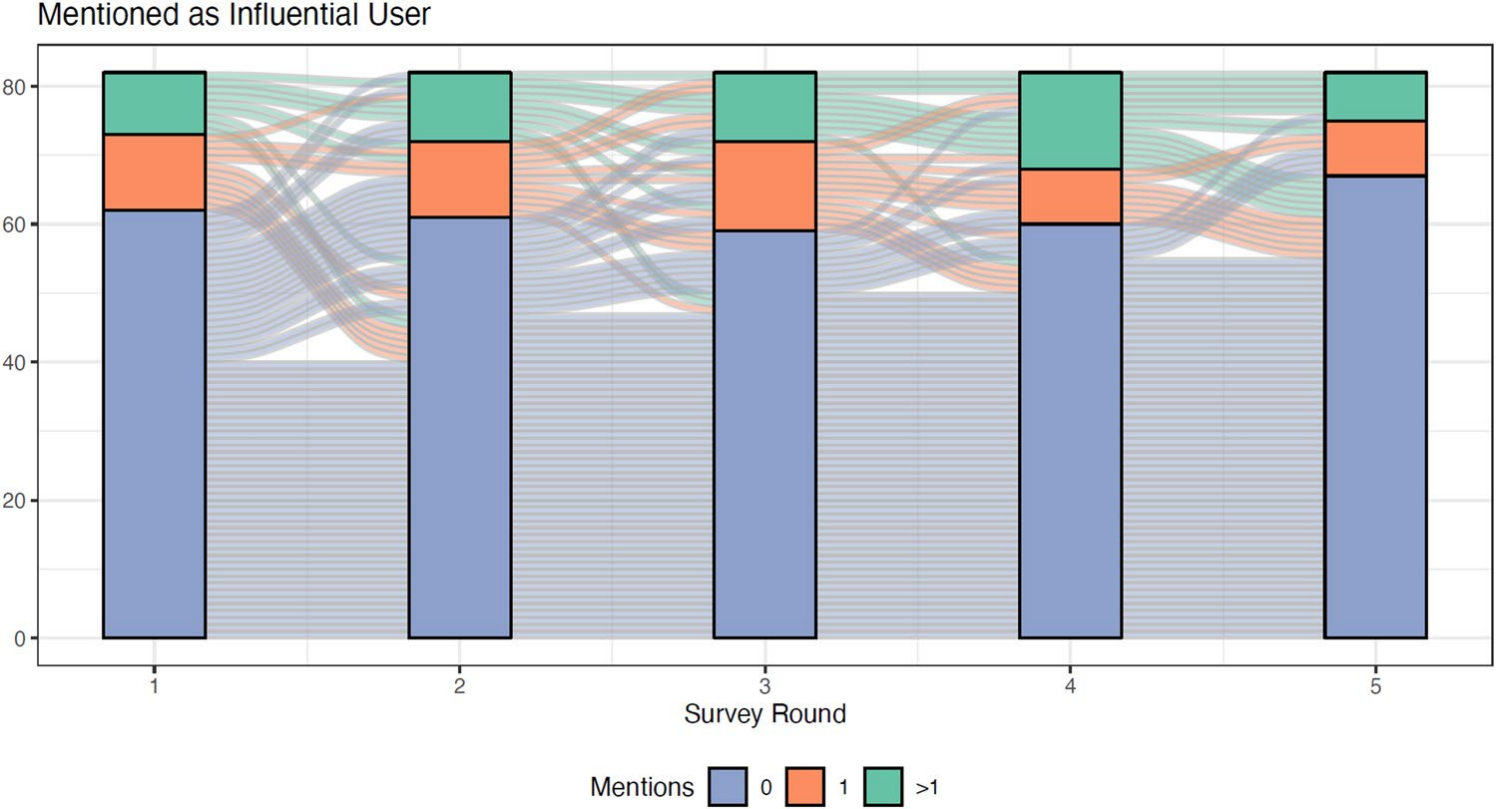
Alluvial plots showing how accounts deemed influential at the beginning tend to stay influential over time. The alluvial flow in this chart shows that most node that were influential at the beginning tend to stay near the top—the green flows descend only slightly and never become unimportant (blue). The orange flows (ones that were fairly influential at the beginning) tend to mostly stay flat or move downward but not too much. The blue flows (not influential at the beginning) stay mostly flat though some upward movement can make them moderately influential (orange) but rarely highly influential (green).

As expected, only a small number of accounts were deemed to be influential by observers. 43 accounts of 116 were mentioned a total of 249 times with 12 of them garnering more than 10 mentions each). Figure 4 shows that those accounts which were not deemed influential at the beginning of our study stayed largely un-influential throughout the study while those that were influential at the end of the study were likely to have been thought of as influential from early on. In fact, of the accounts not mentioned on the first day, 65% were never mentioned even once as being influential during the entire simulation.

While the results clearly show that accounts deemed influential early on tend to stay influential over time, it is not clear whether this confers a first mover advantage, namely that accounts that moved quickly and aggressively in order to gain influence at the beginning tended to project that influence over time. Our simulation was not designed to investigate this hypothesis, but it could be explored in a future simulation.

## Discussion

Given the large and rapidly expanding importance of social media in the communication of information and the formation of social connections, understanding how social media shapes users’ opinions and behaviors is essential for a well-functioning democracy. The lack of critical data about social media use and the opinions of social media users, however, has made it extremely difficult to study influence on social media.

In this paper, we have proposed novel models of influence for online social networks and developed new techniques for evaluating and refining them. Using a custom-built social media platform, we are able to record all aspects of user behavior and, with embedded daily surveys, develop ground truth measures of influence. We show that our models can capture the dynamics of online social influence reliably. Notably, we show that a complete model of influence requires taking into account impressions, in addition to expressions. These findings have important implications for current models of influence that mainly rely on expression data.

Our methods allow us to identify the determinants of influence; i.e., which factors are most predictive of perceived influence. Previous studies have explored these determinants of influence using measures that rely on expressions^25^, but our simulation allowed us to measure perceived influence directly through repeated user surveys. Although we find that expressions and impressions are each associated with influence individually, when estimated simultaneously in the same model, only impressions are statistically significant predictors of influence. These findings highlight the importance of collecting impression data when studying influence in social networks.

Our approach also allows us to study the dynamics of social media behavior and opinion formation in new ways. For example, the data generated from our simulation reveals that users quickly coalesce around a small set of highly influential accounts, with few new accounts achieving influence with the passage of time. Because our simulation allowed us to observe the evolution of online social networks starting from a blank slate with no connections at all, we were also able to capture the formation of these networks in ways that would be impossible using data from preexisting public social media sites. Our data show that although participants initially explored a variety of different accounts and issue positions on the subjects being discussed on the platform, most quickly settled on accounts representing a more homogeneous set of opinions, limiting their exposure to opposing views. These findings may help shed light on the formation of opinion echo chambers on social media.

There are several important limitations to our study. First, the “in-vitro” nature of the simulation can have an effect on the behavior of the participants. Though our users confirmed that our simulation faithfully replicated the environment of prominent social media platforms, our participants’ motivations were primarily monetary, whereas social media users in the real world are more likely to be a mixture of social and political. Likewise, because our participants did not reflect a representative sample of social media users, it is possible that their behavior might differ in important ways from users in the real world, particularly very high-profile users who we might expect to have the greatest influence. Second, our simulation lasted only 5 days. This was long enough to produce valuable data, but not long enough to capture dynamics of influence that may take longer to develop. Third, the “natural” environment in a social media platform is saturated with millions of posts discussing a large variety of topics, while in our simulation the platform was not pre-populated with any posts. Finally, our current measure of influence is not disaggregated by topic. While we did ask users to supply information on the topics they were influenced on, few users opted to answer this voluntary question. As there may be meaningful relationships between influence and topic, future work could extend this experiment by requiring users to provide information about the topic or topics they were influenced on.

Despite these limitations we believe our study offers a number of advantages. Numerous experimental studies in the fields of sociology, psychology, communications, and political science have utilized simulated social media content to explore the dynamics of human behavior and opinion formation on social media^26–28^. Typically, these studies simply expose subjects to a single mock post or news article from a social media site or using simple online games. Our approach significantly improves upon these studies by providing a more realistic environment in which users can interact with each other and react to user-generated content in a more natural way. Because social media platforms do not make impressions data publicly available and because researchers cannot control many critical aspects of the social media platforms they study, simulations like the DartPost-enabled methods in this study provide a valuable tool for researchers. Future iterations of this experiment could further increase the realism of the simulation by increasing the number of users, the length of the experiment, and refining the functionality of the platform.

DartPost also opens up a wide range of avenues for further research. For example, researchers can use the platform to study how humans and social media bots interact, and how different types of bots or levels of bot activity shape network formation and user opinions. Researchers can also explore how disinformation on social media affects users, identifying the conditions that make it more likely to succeed or fail, and whether high levels of misinformation might cause users to distrust even factual information they encounter on the platform. Finally, DartPost may be a useful setting to apply existing methodology in experimental settings to evaluate additional measures of influence^29^.

## Methods

All experimental protocols were approved by the Committee for the Protection of Human Subjects (CPHS) at Dartmouth College. All experiments were performed in accordance with these guidelines and regulations. Informed consent was obtained from all participants.

### Simulation design

To explore social influence in the online environment, we used a separately designed micro-blogging social media platform called DartPost. DartPost mimics many of the main functions of social media platforms like Twitter and Facebook. Unlike commercial platforms, however, DartPost allows researchers to conduct controlled simulations and experiments and to capture a complete record of user behaviors, while maintaining subject anonymity and obtaining positive consent in accordance with human subjects protocols.

DartPost users can post short messages, links and images. Other users can then follow, repost, like or comment on those posts. Users can also tag posts with key words and search  for other posts. The 30 most viewed posts are displayed on the platform homepage. A more detailed description of the platform is provided in the supplementary material 1.

### Experimental design

In this study, we recruited 287 users from Amazon’s Mechanical Turk crowdsourcing service to participate in a 5 day long study. Only American citizens over the age of 18 were included. Mechanical Turk workers were invited to participate in “a social media simulation as part of an academic study of social media usage” and asked to use the platform for between 15 min and 1 h per day, depending on their role in the simulation. Users who consented to participate in the study provided us with their Mechanical Turk worker number, which is not linked publicly to names or other identifying information. Each user was then assigned to one of three main roles: 160 were assigned to be single account operators, 40 were assigned to be multiple account operators and 87 were assigned to be observers. Multiple account operators were then randomly assigned a number of unique accounts between 4 and 8. We aimed to recruit 300 participants, 200 users, and 100 observers, but only successfully recruited 287. We elected to decrease the number of observes to 87 to ensure there was enough content generated on the site. Users did not know the nature or distribution of these roles across other users, or that some other users were operating multiple accounts. Participants were paid 10 USD per hour.

Single and multiple account users were each assigned positions on three of the following eight contemporary political questions: (1) whether the U.S. government had done too much or not enough to combat COVID-19; (2) whether the government should have more authority to regulate social platforms such as Twitter and Facebook; (3) whether the environment or the economy should be given priority in environmental policy; (4) whether they approved or disapproved of the “Medicare for all who want it” health care system; (5) whether they approved or disapproved of a 2% wealth tax on people with more than 50 million dollars in assets; (6) whether they believed that foods containing genetically modified ingredients are safe and healthy to eat; (7) whether they agreed that the United States should pay less attention to problems overseas and concentrate on problems at home; and (8) whether they favored or opposed an increase in the number of nuclear power plants in the United States to provide electricity. Users were instructed that “your task is to get other users to view and like your posts and to convert them to your positions on the issues. You may do this by writing posts or posting links to relevant content on the Web, reposting other users’ content on DartPost, or replying to or liking other posts”. To incentivize users to actively seek to influence other participants, users were informed that they would be given a bonus payment that would increase “the more users who view, like, and repost your posts, and the more users you convince to support your issue positions”.

Observers were assigned passive accounts. These accounts had the capability to follow other users, but could not like, post, repost, or comment. Unlike the other users, observers were not assigned positions on any of the eight issues. Observers’ instructions stated “Your job is simply to observe what other users are doing on the platform. These users are trying to maximize positive exposure and agreement with the positions they support on several key political and social issues...”.

Before each user was assigned a role at the beginning of the simulation, and then once each 24 h after completing their minimum time using the platform, all users were asked to complete a survey. The initial survey collected standard demographic data including age, race, gender, educational attainment, political affiliation, and information about the subject’s use of social media. All subjects were then asked to indicate their position on the eight political questions described above. On each subsequent day, users were asked the same eight questions again, allowing us to track changes in opinions over the course of the simulation. The initial and the daily survey questions, more information on the participants, and statistics about the platform usage during the experiment are shown in the supplementary material 1.

### Subject and polarity annotation

The subject and polarity of all the posts and comments were manually annotated by four Dartmouth undergraduate students. The annotators were asked to categorize the texts into nine subject categories (the eight aforementioned political topics and a miscellaneous category) and three polarity categories (positive, negative, neutral). Majority voting (i.e., agreement between at least three of the four annotators) was used to come up with a final label for each post and comment. There was majority agreement for 96% and 98% of the subject and polarity labels, respectively.

## Supplementary Information

Supplementary Information.

## Data Availability

The code and data for replicating the results presented in this paper has been made available to the reviewers as a supplementary ZIP file. These files will be made publicly available after publication at https://github.com/ruiliu310/dartpost-research/tree/master/open.
