# Supplementary material for: Using impression data to improve models of online social influence
Source: Sci Rep. 2021 Aug 16;11:16613. doi: 10.1038/s41598-021-96021-3 (PMC8368058; doi:10.1038/s41598-021-96021-3)
Supplement: Supplementary file 1 — Supplementary Information. [file 41598_2021_96021_MOESM1_ESM.pdf]

Supplementary Information: Using Impression Data to Improve Models of Online Social Influence

Rui Liu[1], Kevin T. Greene[1], Ruibo Liu[1], Mihovil Mandic[1], Benjamin A. Valentino[2], Soroush Vosoughi[1], and V.S. Subrahmanian[1,*]

[1]Dartmouth College, Department of Computer Science, Hanover, 03755, USA
[2]Dartmouth College, Department of Government, Hanover, 03755, USA
[*]vs@dartmouth.edu

ABSTRACT

Influence, the ability to change the beliefs and behaviors of others, is the main currency on social media. Extant studies of influence on social media, however, are limited by publicly available data that record expressions (active engagement of users with content, such as likes and comments), but neglect impressions (exposure to content, such as views) and lack “ground truth” measures of influence. To overcome these limitations, we implemented a social media simulation using an original, web-based micro-blogging platform. We propose three influence models, leveraging expressions and impressions to create a more complete picture of social influence. We demonstrate that impressions are much more important drivers of influence than expressions, and our models accurately identify the most influential accounts in our simulation. Impressions data also allow us to better understand important social media dynamics, including the emergence of small numbers of influential accounts and the formation of opinion echo chambers.

DartPost Platform

DartPost was designed to mimic many of main functions of social media platforms like Twitter and Facebook, while giving us fine-grained insight and control over the actions of all users. Here, we briefly go over all the main functionalities afforded to users and experimenters by our platform.

Account Creation & User Profile

DartPost allows users to create accounts using an email address. For the purpose of our experiment, we created all the accounts before the start of the experiment and assigned them to the participants. The accounts were blank and the participants were able to personalize their account and profile. Figure S1 shows an example landing page of a user.

Social Network

Similar to Twitter, DartPost includes a directed (i.e., followers and followees) social network. Users can follow and unfollow each other through their profile. Figure S2 shows the follow/unfollow button.

User Timeline

A user’s timeline is a scrollable collection of the posts by the followees of that user (i.e., the accounts the user follows) in reverse chronological order. Figure S2 shows an example timeline on DartPost .

Posting & Reposting

Posting

Similar to mainstream social media platforms, such as Twitter, and Facebook, users on DartPost can post short text messages. The messages can contain urls to external sources (such as news websites) and can have images embedded in them. Also, similar to most social media platforms, posts can be tagged with a topic. Similar to other platforms, the tags on a post appear with a ’#’. Figure S3 shows the interface for posting (note the options for including tags, urls, and images).

Reposting

Similar to the Retweet functionality, posts can be reposted by other users. Reposting has the effect of increasing its likelihood to appear in the trending posts section. Reposting also pushes a message to the timeline of the followers of a user. There is a counter, visible to all, that shows the total number of times a post has been reposted.

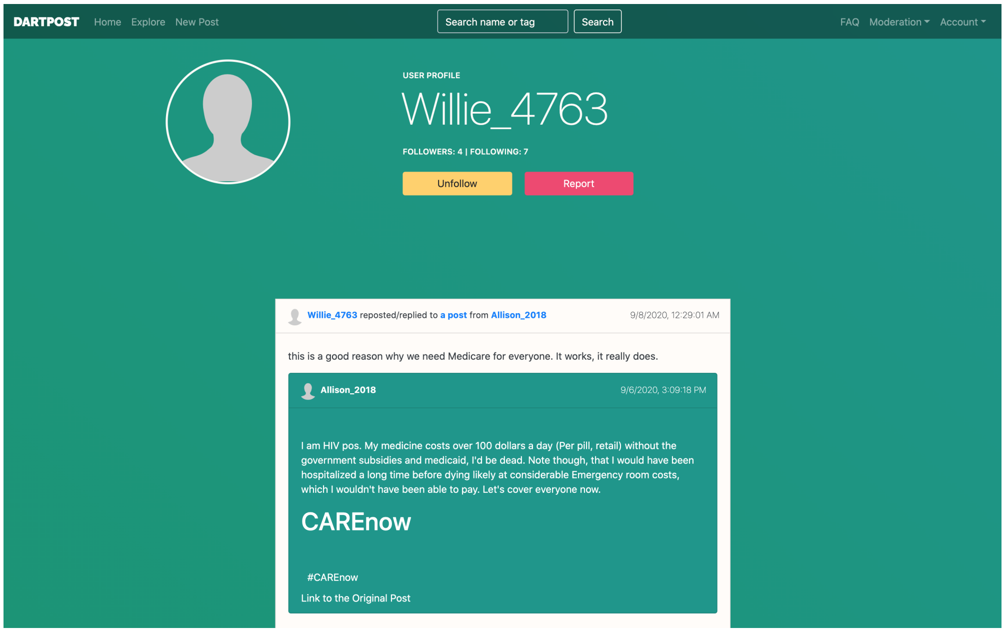

Figure S1. Example of a user's profile on DartPost .

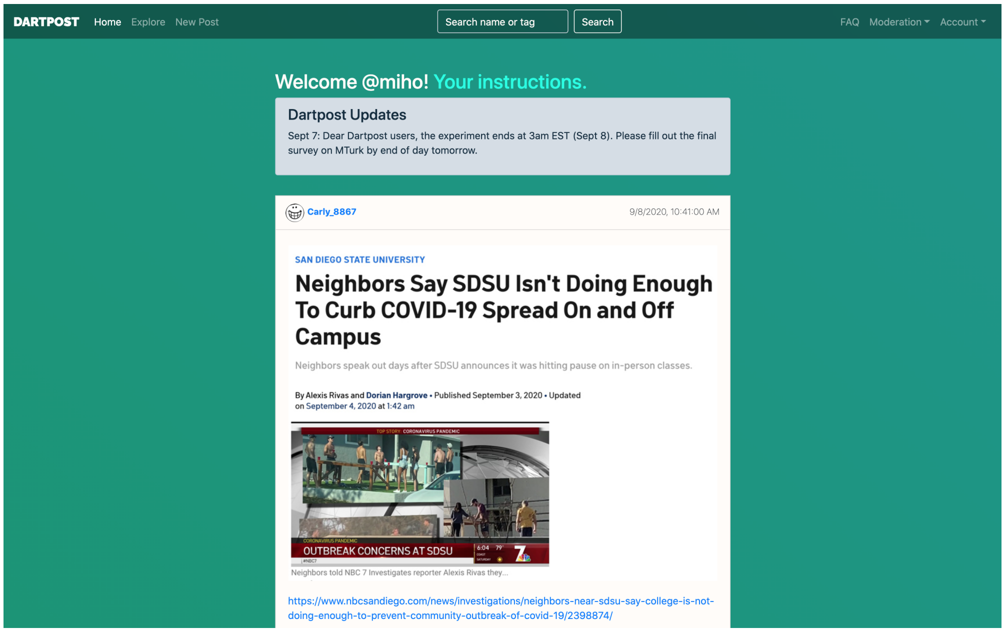

Figure S2. Example of a user's timeline on DartPost . Here, we are showing the top message in that user's timeline.

Replying

Similar to Twitter's quote functionality, users can embed another post in their posts, thus allowing them to directly address the point raised in the original post. Figure S4 shows an example reply.

Commenting

Users can also comment on other people's posts. This allows users to engage in a discussion around particular issues. Like reposting, comments also increase the likelihood of a post to appear in the trending posts section of the platform. Figure S5 shows an example thread of comments.

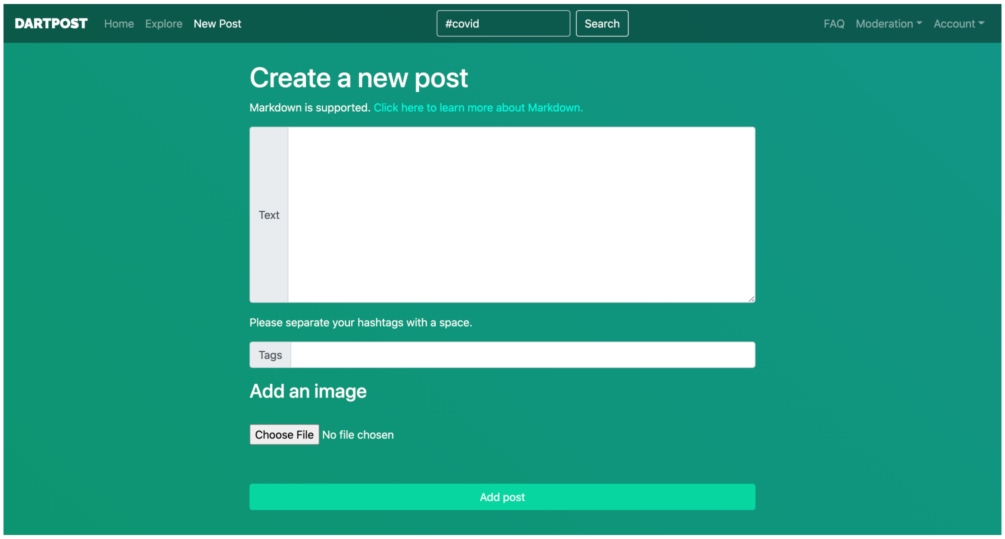

Figure S3. Posting on DartPost .

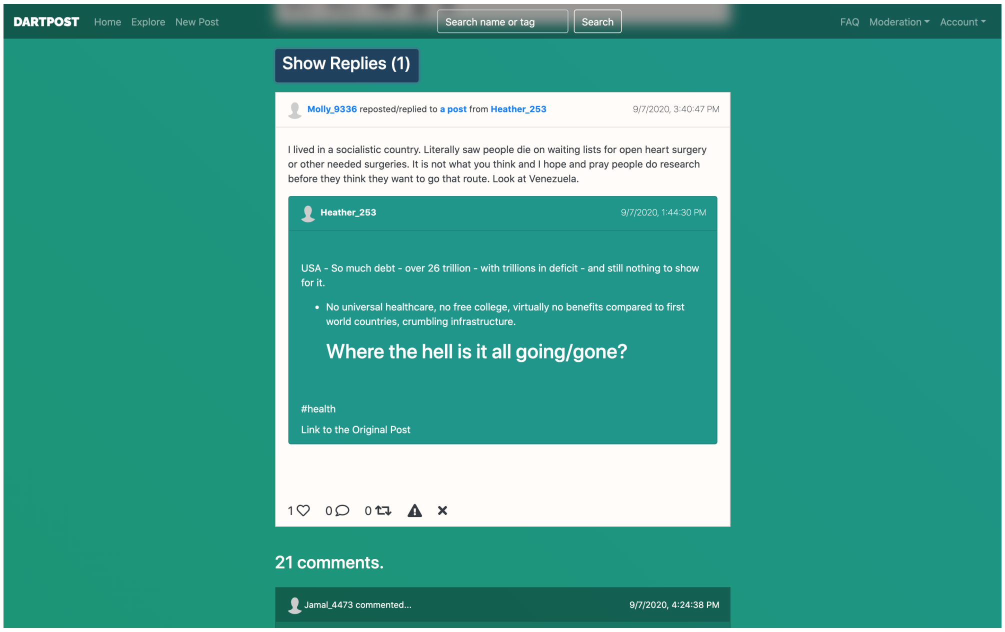

Figure S4. Replying on DartPost .

Liking

Similar to most commercial platforms, users can "like" posts. As with reposting, there is a counter, visible to all, that shows the total number of likes for a post.

Search

DartPost also includes basic search functionality that allows users to search for specific topics or users. Tags are used for the topic search, while usernames are used for the user search. Figures S6 and S7 show examples of the search functionality on tags and usernames, respectively.

Trending Posts

DartPost includes a page where participants could explore the trending posts on the platform. Trending post scores where calculated using the following formula:

$$\text{Trending\_Score} = \#\text{ of likes} + \#\text{ of reposts} \times 0.75 + \#\text{ of comments} \times 0.5$$

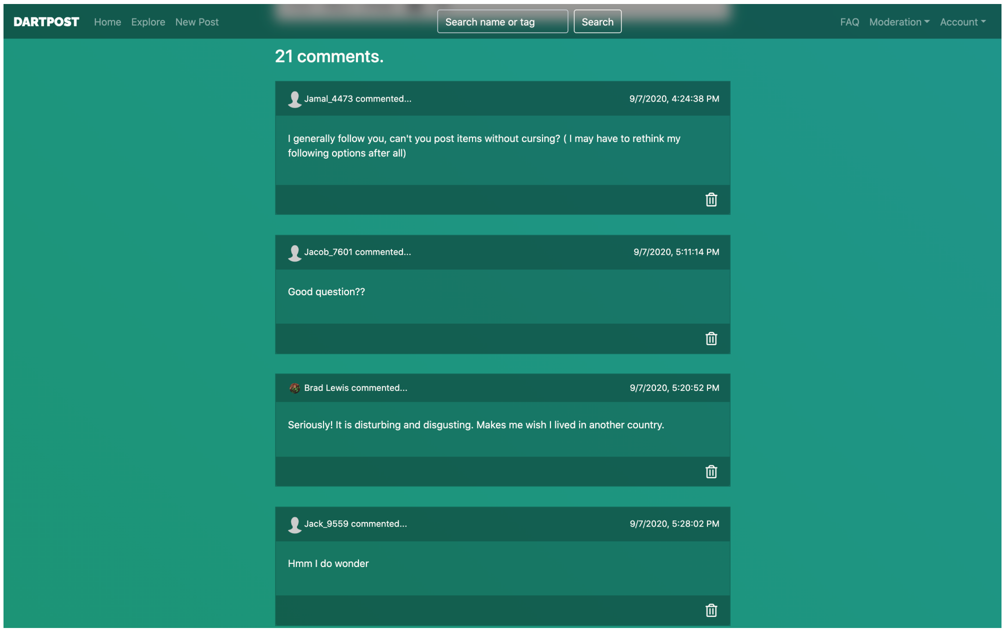

Figure S5. Commenting on DartPost .

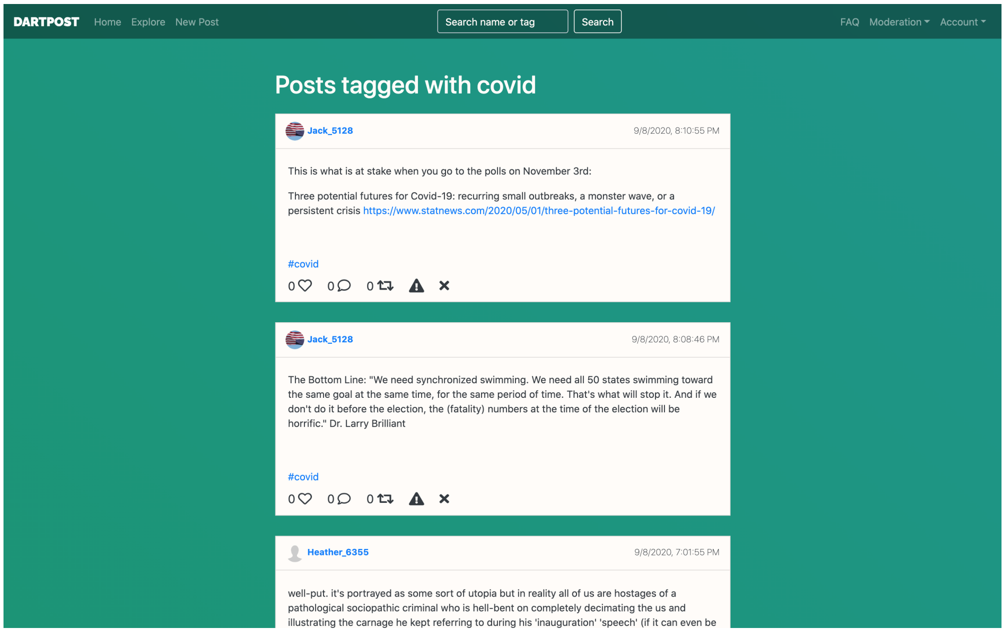

Figure S6. Example of tag search on DartPost Here, posts containing the tag #covid are shown.

The trending posts page shows the top 30 posts within the last 8 hours using the formula above.

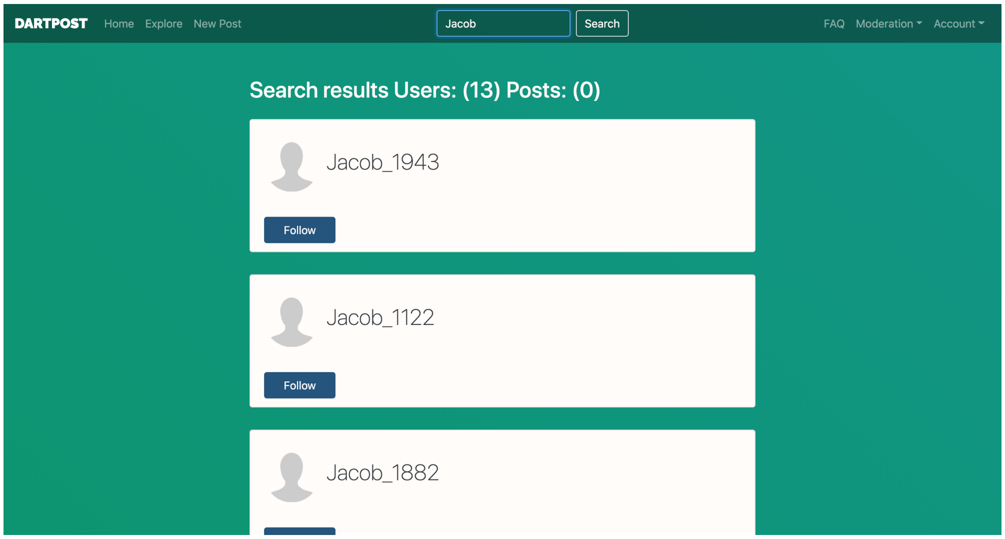

Figure S7. Example of username search on DartPost . Here, the user has searched for the name "Jacob". All usernames containing "Jacob" are returned.

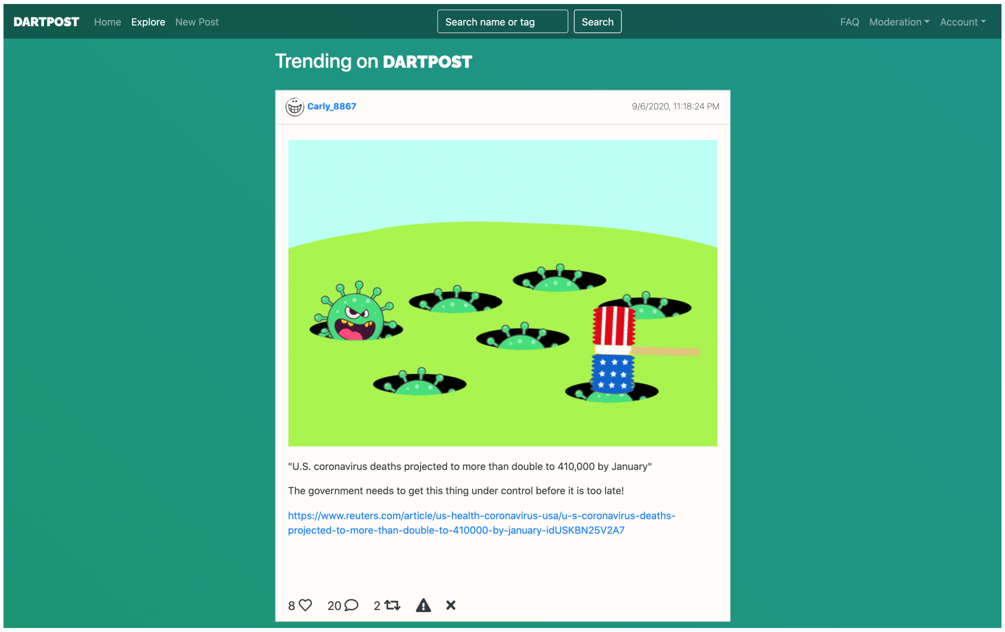

Figure S8. The explore/trending page on DartPost . The most recent and most popular posts are shown on top. Here, the top post is shown, with 8 likes, 20 comments, and 2 reposts.

Impression Data

Unlike commercial platforms, DartPost allows us full access to impression data. To gather post impression data, we created a database model called postView. Every time a post is displayed to a user, we record where and when it was displayed. There are five certain types of post views that we keep track of:

- A timeline view is recorded when a user views a post on their private timeline, a page where they can view the most recent posts from other users they chose to follow.
- An explore view is recorded when a user views a post on the explore page, a page that shows 30 trending posts created within the last 8 hours.
- A search view is recorded when a user searches for a hashtag and the post came up in the results.

- A user page view is recorded when a user views a post on someone's user profile.
- Finally, the last type of impression is the individual post view, and it is recorded when a user clicks on a specific post to read its comments or more about it. The individual post views are the most direct impression data, as we know that a user certainly had to see the post after clicking on it.

Expression Data

The expression data in DartPost corresponds to posts (including reposts and replies) and comments. These are analogous to expression data available to researchers from commercial platforms. For example, on Twitter, one can get access to tweets, quoted tweets, retweets, and replies.

Experimental Detail

Participants

Amazon Mechanical Turk was used to recruit 287 participants. The participants were divided into three roles: (i) Single account operators (160) (ii) Multiple account operators (40) (iii) Passive observers (87). The demographic breakdown of all participants are shown in Figure S9.

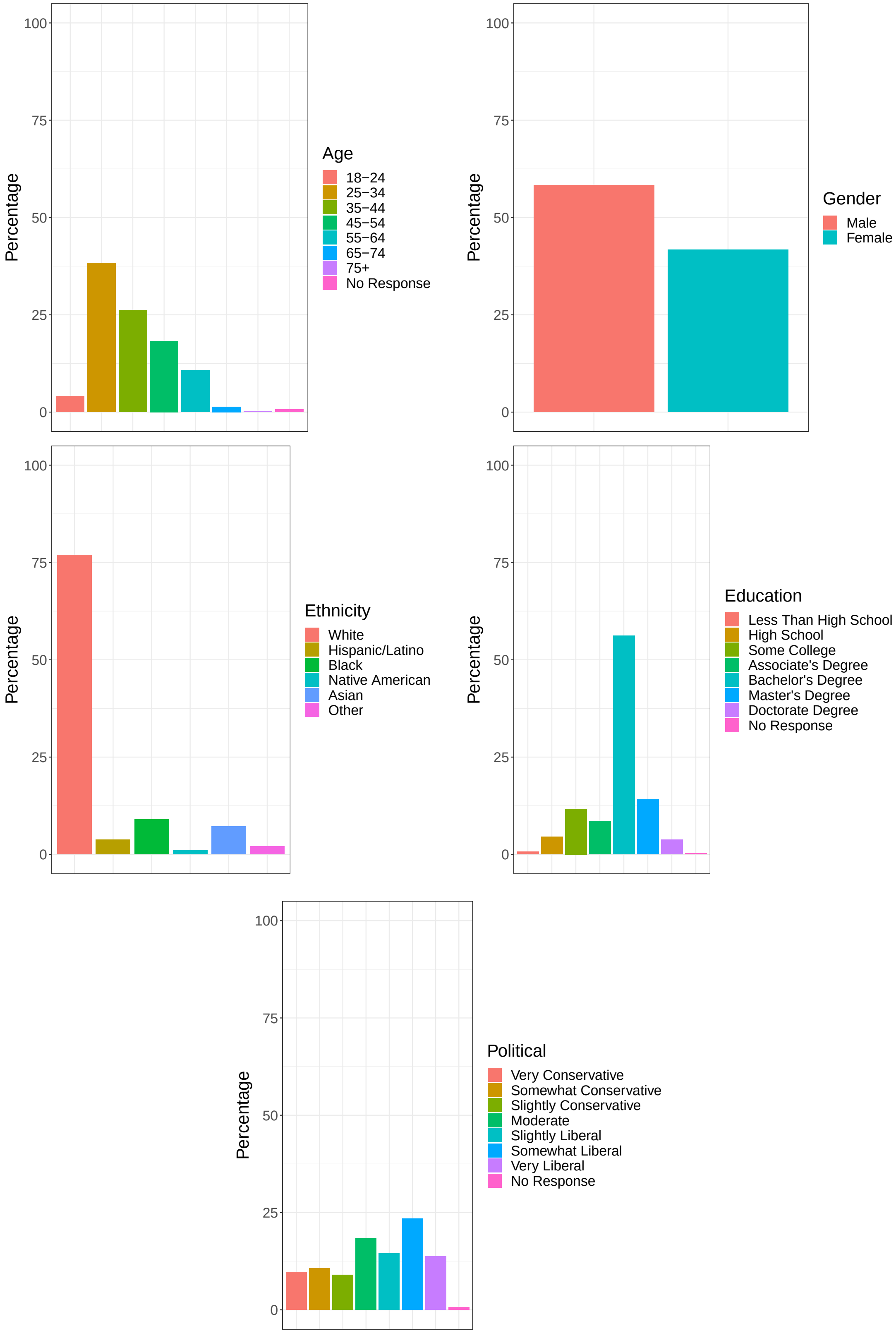

Figure S9. The demographic breakdown of the participants.

Surveys

Participants were asked to complete two types of survey, an initial survey given to all participants before instructions were given to them, and a daily survey that the participants were required to complete every 24 hours. The questions for the initial and daily surveys are attached as appendices A and B, respectively.

Initial Survey The initial survey was designed to gauge the demographic and political and ideological position of the participants.

Daily Survey The daily survey was designed to measure the effects of the experiment on the participants. The daily surveys were all identical. Participants were required to show proof of survey completion before they were paid for that day. This was done by providing them with a unique identifier at the end of each survey that they had to enter in a form in Amazon Turk for payment.

Damping	*K*								
	1	5	10	20	30	40	50	100	116
0.85	0.196	0.609	0.772	0.783	0.794	0.794	0.794	0.794	1.000
0.90	0.239	0.598	0.772	0.783	0.794	0.794	0.794	0.794	1.000
0.70	0.196	0.609	0.772	0.783	0.794	0.794	0.794	0.794	1.000
0.50	0.207	0.620	0.772	0.783	0.794	0.794	0.794	0.794	1.000
0.30	0.228	0.620	0.772	0.783	0.794	0.794	0.794	0.794	1.000
0.10	0.239	0.630	0.772	0.783	0.794	0.794	0.794	0.794	1.000

Table S1. Full precision at K results for FI_1.

K								
1	5	10	20	30	40	50	100	116
0.1957	0.3478	0.7065	0.7609	0.7935	0.7935	0.7935	0.7935	1.0000

Table S2. Full precision at K results for FI_2.

Effectiveness of FI_1 and FI_2

Tables S1 and Tables S2 shows the results of our precision at K assessments for FI_1 and FI_2 respectively. Note that for FI_2, there is no damping factor and as such no search was conducted. Similar to FI_3, the FI_1 is not sensitive to the choice of the damping factor, especially at higher Ks.

Impression vs. Expressions

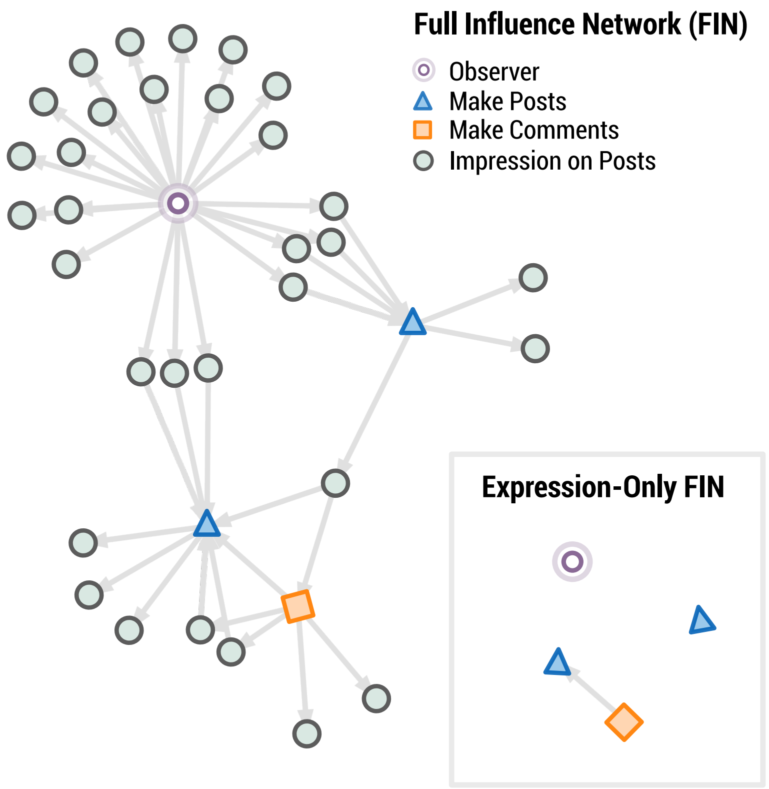

Figure S10. Sample Full Influence Network using both expression and impressions and expressions only (inset) for a single observer at a particular day and subject-polarity pair (this node is shown in purple). Different shapes indicate different types of events – impression events (post served to user or comment served to user) are shown as circles, Making Comments are shown as boxes, while Making Posts are shown as triangles.

Figure S10 depicts an example based on data from our simulation of a full FIN and a FIN that is restricted to expression events only for the same influence event. It is clear that the expression only model provides a much sparser picture of user interactions than the model including impressions. In our Expression-Only example there is a single edge between a post and a comment on that post. Because observers in our simulation can not make posts or comments, there are no edges from the observer to any of the expression data. However, when we include impression information, we now see edges between the expression data and the observer. Further, when we expand to the FIN we see how impression can form a more complete network when we extend out to include impressions on comments and posts.

User Activity Over Time

Figure S11 displays the average participant activity for each day of the simulation. For each day in the simulation, we display the mean number of posts and comments made, and the number of views received. To present some uncertainly around this estimate we also display the interval two standard errors from the mean as dotted lines. We present this information both for users that were mentioned as being influential and those that were not. Influential users tend to have higher average values of comments made, posts made, and views received across most days in the simulation. However, consistent with the regression results in the main paper, the number of views received by influential users are consistently larger than non-influential users. While this is expected, it further reinforces the importance of having access to impression data (views) not only expression data (comments/posts) when attempting to measures online influence.

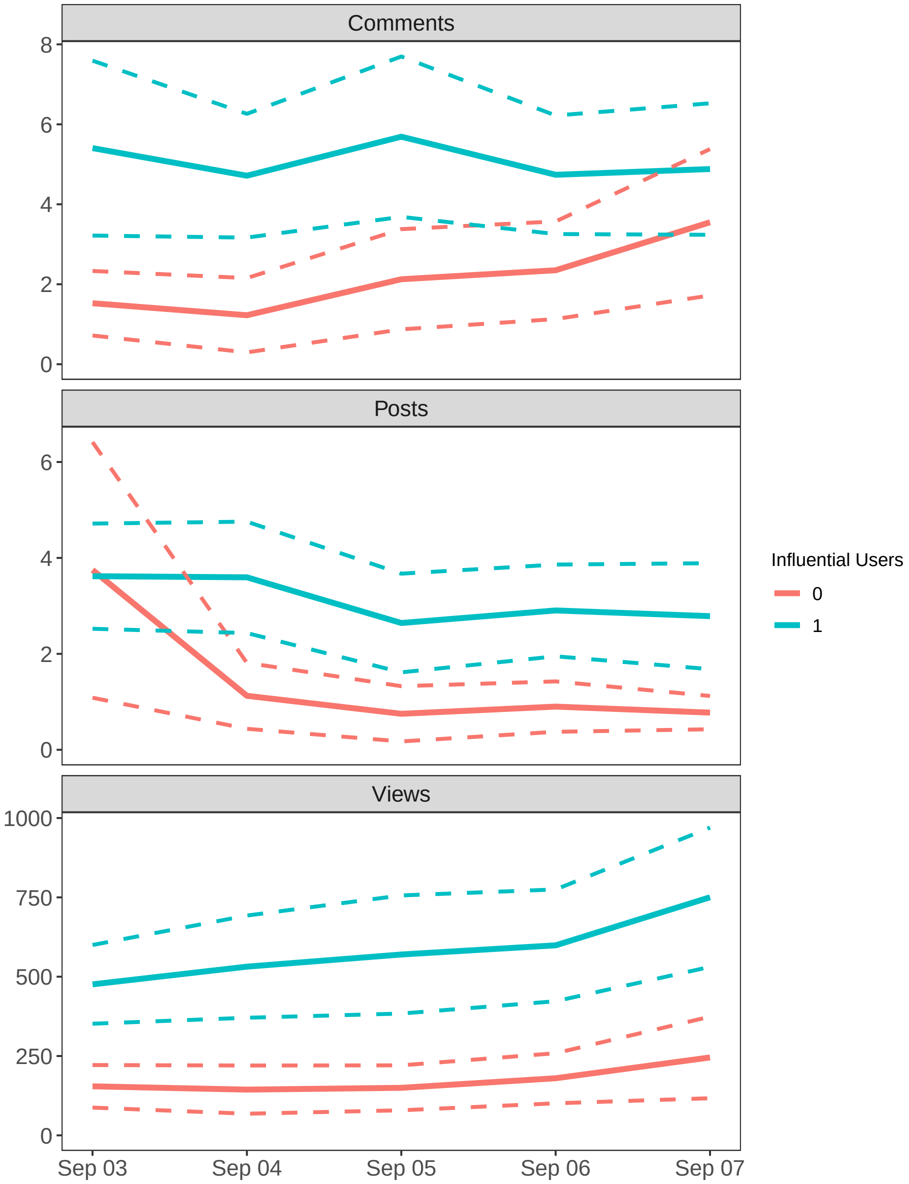

Figure S11. Average daily activity among participants. The solid lines denote mean values for each measure, while the dotted lines represent values two standard errors from the mean. We present average activity for influential users (blue) and non-influential users (red).
